# Using proximity extension proteomics assay to discover novel biomarkers associated with circulating leptin levels in patients with type 2 diabetes

**DOI:** 10.1038/s41598-020-69473-2

**Published:** 2020-08-04

**Authors:** Camilla Vavruch, C. Nowak, T. Feldreich, C. J. Östgren, J. Sundström, S. Söderberg, L. Lind, F. Nyström, J. Ärnlöv

**Affiliations:** 1 Department of Medical and Health Sciences, Linköping University, Linköping, Sweden; 2 Family Medicine and Primary Care Unit, Department of Neurobiology, Care Sciences and Society (NVS), Karolinska Institutet, Huddinge, Sweden; 3 School of Health and Social Studies, Dalarna University, Falun, Sweden; 4 Department of Medical Sciences, Uppsala University, Uppsala, Sweden; 5 Department of Public Health and Clinical Medicine, Section of Medicine, Umeå University, Umeå, Sweden

**Keywords:** Cardiovascular diseases, Predictive markers, Diabetes

## Abstract

We aimed to discover novel associations between leptin and circulating proteins which could link leptin to the development of cardiovascular disease in patients with type 2 diabetes (T2DM). In a discovery phase, we investigated associations between 88 plasma proteins, assessed with a proximity extension assay, and plasma leptin in a cohort of middle-aged patients with T2DM. Associations passing the significance threshold of a False discovery rate of 5% (corresponding to *p* < 0.0017) were replicated in patients with T2DM in an independent cohort. We also investigated if proteins mediated the longitudinal association between plasma leptin and the incidence of major cardiovascular events (MACE). One protein, adipocyte fatty acid binding protein (A-FABP), was significantly associated with leptin in both the discovery phase [95% CI (0.06, 0.17) p = 0.00002] and the replication cohort [95% CI (0.12, 0.39) p = 0.0003]. Multiplicative interaction analyses in the two cohorts suggest a stronger association between A-FABP and leptin in men than in women. In longitudinal analyses, the association between leptin and MACE was slightly attenuated after adding A-FABP to the multivariate model. Our analysis identified a consistent association between leptin and A-FABP in two independent cohorts of patients with T2DM, particularly in men.

**Trial registration**: ClinicalTrials.gov identifier NCT 01049737.

## Introduction

Coronary heart disease (CHD) and cerebrovascular disease are leading causes of death worldwide and together they are called cardiovascular disease (CVD)^1^. An important risk factor for CVD is type 2 diabetes mellitus and there is an urgent need for clinically useful risk markers for CVD in type 2 diabetes in order to identify high-risk individuals who could benefit from targeted preventive measures. Leptin is a hormone/cytokine secreted by adipose tissue^2^. Circulating leptin levels reflect the amount of adipose tissue in the body, and leptin regulates body weight by acting in the hypothalamus to increase energy expenditure, and suppress food intake and appetite^3^. Impaired leptin signaling induces severe obesity in mice^4^ and humans^5,6^. The feedback loop to the central nervous system regulating adipose tissue size by leptin signaling is influenced by so called “leptin resistance”^7,8^. Previous studies have shown an independent link between circulating leptin levels and the development of CVD^9–11^, and our previous study confirmed these results^12^. Leptin is also produced by lymphocytes and can promote the activation of monocyte-macrophages^13,14^. Exactly how leptin is involved in inflammation and CVD is not yet fully known. Leptin-associated inflammatory and cardiovascular biomarkers could provide further biological insights and potentially lead to new drug targets or interventions in the future.

The concurrent measurement of a large number of proteins in biological samples (proteomics) has evolved as an important research field in the last decades, with a hope that it would be useful for identifying candidate biomarkers and novel mechanisms for disease^15^. In the present study, we hypothesized that circulating proteins could be mediators that link leptin with the development of cardiovascular disease. Therefore, we aimed to discover and replicate the association between plasma leptin and 88 proteins from a multiplex proteomics assay in two independent cohorts of type 2 diabetes patients. In addition, we aimed to explore if these proteins mediate the association between plasma leptin and future cardiovascular events.

## Methods

### CARDIPP

For the discovery analysis, we used baseline data from 661 individuals participating in the community-based cohort CArdiovascular Risk factors in patients with Diabetes—a Prospective study in Primary care (CARDIPP, https://clinicaltrials.gov/ct2/show/NCT01049737) that had data on plasma leptin, proteomics and all co-variates. An in-depth description of the study has been presented before^12,16^. In short, participants were consecutively recruited from November 2005 to 2008 from 22 different primary health care providers in the counties of Östergötland and Jönköping in Sweden. All participants had type 2 diabetes mellitus and were 55–66 years old at time of recruitment. Primary health care centers were selected to represent different demographic, large- and small-intake, rural and urban areas, and the health care centers differed in size. The model of treatment and care was organized similarly and adhered to the same national diabetes care guidelines. Follow-up assessment was carried out in 2014.

At baseline, a nurse dedicated to the treatment of diabetes at the primary health care centers measured height and weight, and a standardized medical history was taken. Blood was drawn in the morning after a 10-h over-night fast and was analyzed for standard tests such as plasma glucose and serum lipids routines at the primary health care centres. Blood samples were spun down and stored as EDTA plasma samples at – 70 °C until analysis. Glomerular filtration rate was estimated using the creatinine based CKD-EPI equation^17^.

### PIVUS

For replication, a longitudinal prospective study, Prospective Investigation of the Vasculature in Uppsala Seniors (PIVUS, https://snd.gu.se/sv/catalogue/study/ext0162), was used, which has been reported in detail previously^18^. In short, PIVUS started in 2001 when a non-selective sample of 70-year-old Uppsala community residents were recruited to evaluate measures of endothelial function. Of these, 116 had diabetes mellitus type 2, all of which were included in the present study. In secondary analyses we also investigated whether significant findings from the analyses in individuals with diabetes also were generalizable to individuals without diabetes in PIVUS (n = 876).

### Leptin measurements

In CARDIPP, the Milliplex^®^ MAP Gut Hormone Panel (Merck Millipore, Billerica, MA, USA) designed for analysis with Luminex^®^-technique (Luminex, Austin, TX, USA) was used for analyzing leptin levels. Total intra and inter assay coefficient of variation for leptin was 11%. In PIVUS, plasma leptin levels were analyzed with double antibody radioimmunoassay (RIA) method (Linco Res, St Louis, Missouri, USA). Total variation coefficient for leptin was 4.7% at both low (2–4 ng/mL) and high (10–15 ng/mL) levels.

### Multiplex protein assay

**Figure 1:**
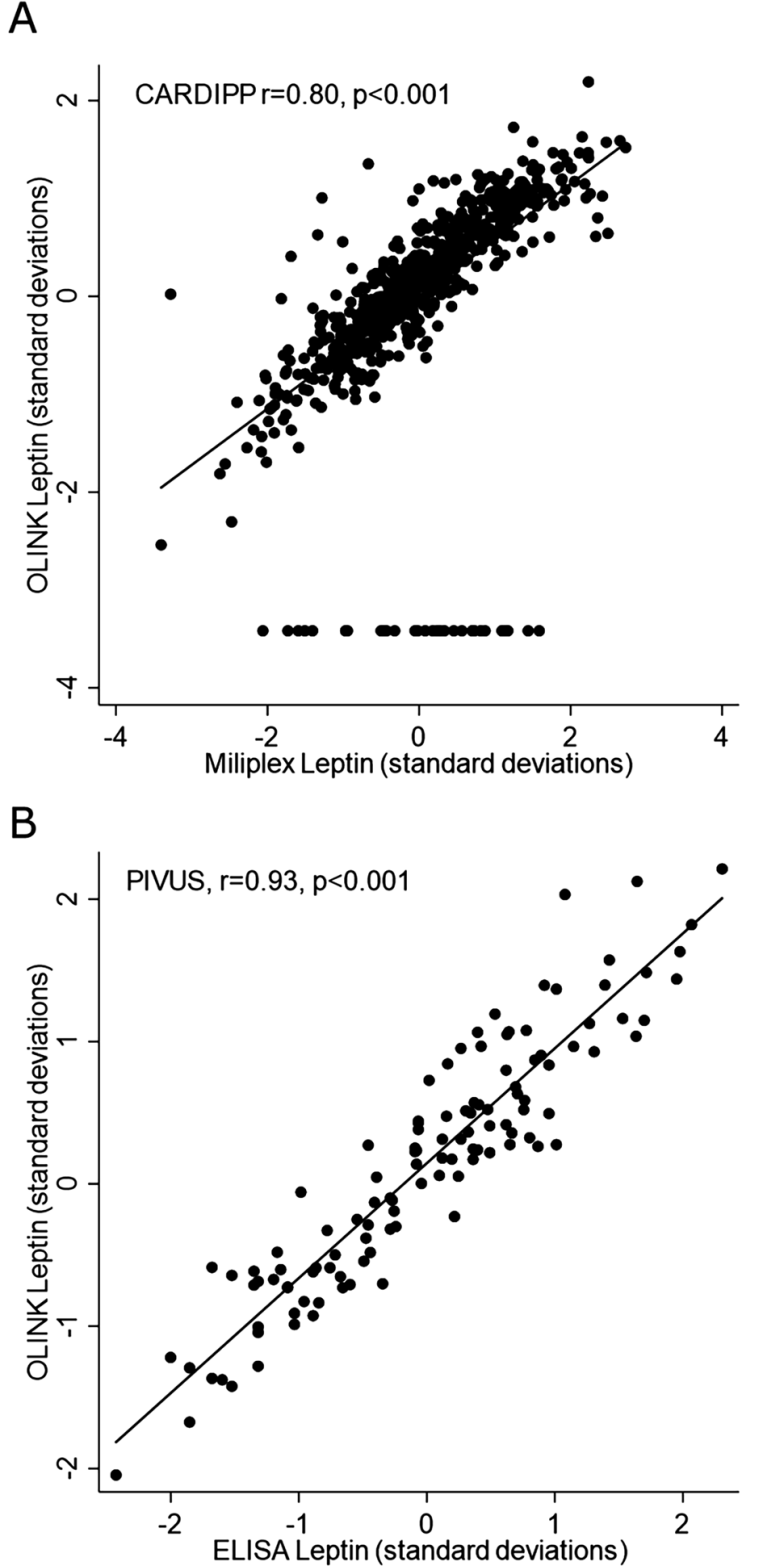
Leptin/leptin sensitivity analysis CARDIPP (**a**) and PIVUS (**b**). Spearman correlation coefficient 0.8 for CARDIPP and 0.93 for PIVUS, p < 0.001 for both.

The Proseek CVD Multiplex 96 × 96 (Olink, Uppsala, Sweden) was used to measure proteins in plasma by real-time polymerase chain reaction. It measures 92 cardiovascular proteins, one negative control and three positive controls for internal control, using the proximity extension assay method. It has been previously used for discovering biomarkers for cardiometabolic traits^19^. The proteomics well plate uses two antibodies for each protein, and a polymerase chain reaction (PCR) step to achieve high-specific binding, allowing for the opportunity for parallel, multiple protein measurements. The technique gives no absolute concentrations of the proteins. The following proteins were excluded because of > 15% missing values: melusin, natriuretic peptides B, and interleukin 4. Proteins with fewer missing values were imputed by the lower limit of detection (LOD) threshold divided by two (LOD/2). We also excluded leptin which was one of the proteins included in the multiplex assay. The leptin measurements by Milliplex^®^ MAP Gut Hormone Panel in CARDIPP and RIA method in PIVUS were closely associated with leptin assessed by the proximity extension assay (Spearman correlation coefficient 0.8 and 0.93, respectively, *p* < 0.001 for both, Fig. 1a,b, respectively). The complete list of included proteins is presented in Supplementary Table 1.

### Study outcome

The CARDIPP participants were followed for occurrence of death or hospitalization for major adverse cardiac events (MACE), which includes cardiovascular death, myocardial infarction, and non-fatal stroke, until December 31, 2014. Data was collected from the Swedish Cause of Death and Inpatient registries (The National Board of Health and Welfare, Stockholm, Sweden) using their unique personal identification numbers.

### Statistical analysis

**Figure 2:**
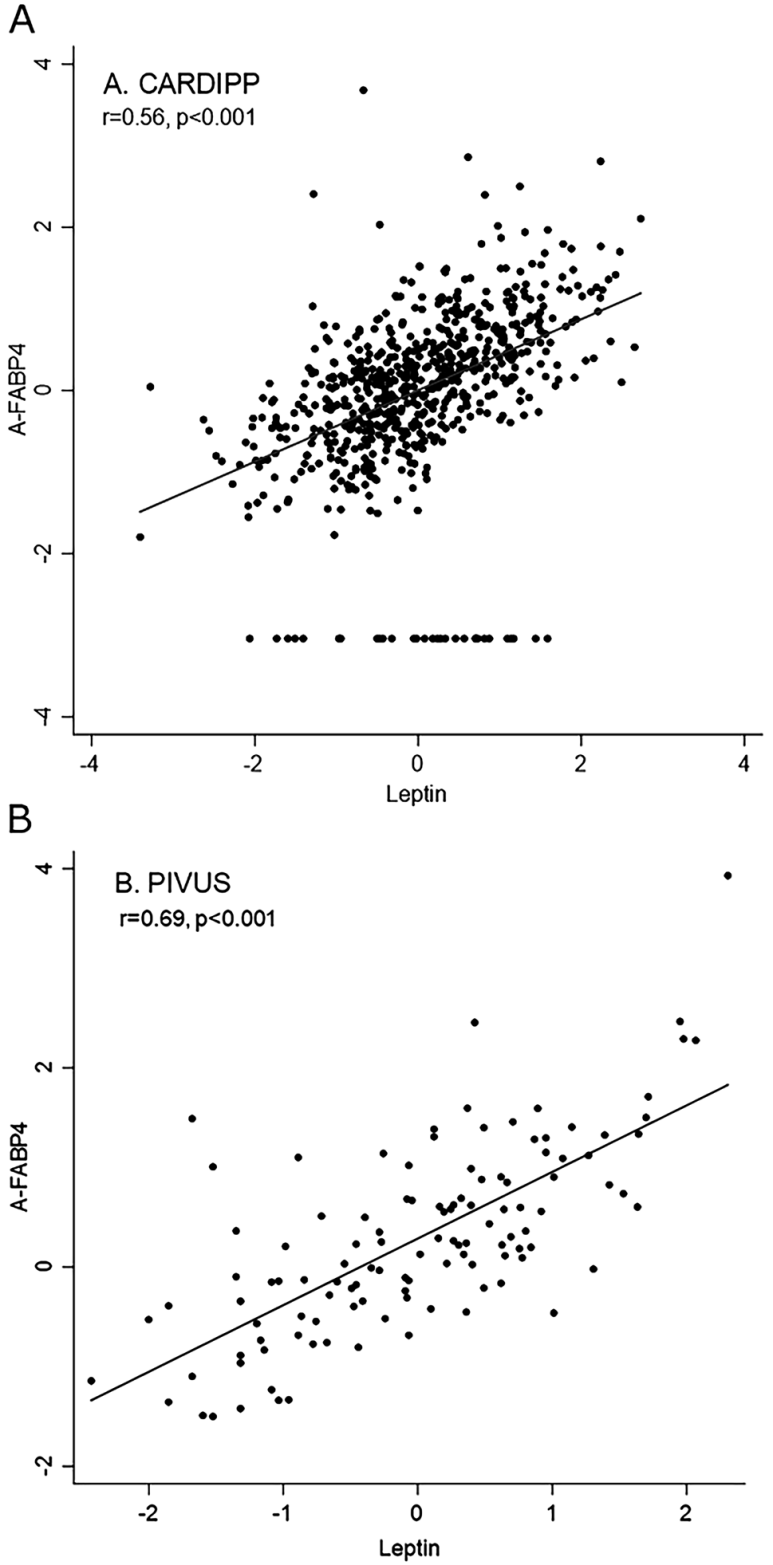
Scatterplot of Spearman correlation coefficient. Spearman correlation coefficient between serum leptin and A-FABP for (**a**) CARDIPP and (**b**) PIVUS. Units are standard deviation for all.

For the primary analysis, CARDIPP was used as the discovery sample and PIVUS for replication. For discovery, separate linear regression models adjusted for age, sex and body mass index (BMI) (as BMI is very closely related to leptin levels and women generally have higher serum leptin levels than men^20^) were performed for each the 88 proteins in order to assess associations between the proteins and plasma leptin. In these analyses both proteins and leptin were scaled to standard deviation units. Proteins associated with leptin at a false discovery rate < 0.05 (corresponding to a p-value of < 0.0017) were taken further to linear regression analysis in the replication cohort PIVUS. In the replication step, we considered nominal p-values (*p* < 0.05) to be statistically significant. We performed additional multivariable analyses for proteins that were consistently associated in both cohorts adjusting for age, sex, BMI, systolic blood pressure, low density lipoprotein (LDL) cholesterol, eGFR, and high density lipoprotein (HDL) cholesterol. We also explored models further adjusted for waist circumference and sagittal abdominal diameter. Additionally, we calculated Spearman correlation coefficient between leptin and replicated proteins and depicted these associations in scatter plots (Fig. 2).

Finally, we investigated to what degree the proteins that were cross-sectionally associated with leptin mediated the longitudinal association between leptin and MACE incidence, using multivariable Cox regression adjusted for age, sex, BMI, systolic blood pressure, GFR, LDL and HDL cholesterol with and without the protein.

### Ethics approval

The study protocol complied with the Declaration of Helsinki. CARDIPP was approved by the Regional Ethical Review Board in Linköping, Sweden (Dnr M26-05). PIVUS was approved by the Ethics Committee of the University of Uppsala (Dnr. 00419 and 2005/M-079).

### Consent for publication

All participants gave informed consent prior to participating in the studies. CARDIPP was conducted in 2005–2008, PIVUS started in 2001. For the present article, we used data from both studies that already existed but had not been studied before. The owners of the data from both CARDIPP and PIVUS have all given permission to use the data and are co-authors of the article.

## Results

Baseline characteristics for participants in both cohorts are shown in Table 1. In CARDIPP, serum leptin was successfully measured in 225 women and 436 men in the cohort, with a mean age of 60.7 years (range 54–66) and with a mean BMI of 30.1 kg/m^2^ (range 18.3–51.7). For PIVUS, we had data on 48 women and 68 men (mean age 70.0 years), with a mean BMI of 29.1 (range 18.2–49.8 kg).

**Table 1:** Baseline characteristics.

	Discovery sample CARDIPP	Replication sample PIVUS
n	661	116
% women	34	41
Age, years	60.7 ± 3.1	70.1 ± .1
BMI, kg/m^2^	30.1 ± 4.1	29.1 ± 5.2
LDL-cholesterol, mmol/l	2.7 ± .8	2.9 ± .9
HDL-cholesterol, mmol/l	1.3 ± .3	1.4 ± .4
SBP	137 ± 16	154 ± 24
Leptin, ng/ml	8.3 ± 8.9	14.7 ± 11.3
Leptin, ng/ml—men	5.1 ± 4.7	9.3 ± 5.8
Leptin, ng/ml—women	14.4 ± 11.4	22.4 ± 12.8
eGFR	78.5 ± 14.1	79.2 ± 19.3
Diabetes duration	7.15 ± 6.2	8.3 ± 7.7
% with blood pressure treatment	65.4	56.0
% pharmacological diabetes treatment	71.9	58.6
% with statin treatment	54.4	28.5

Continuous variables are given as mean ± SD, dichotomous variables as n (%).

In the discovery analysis in CARDIPP, two proteins were significantly positively associated with to leptin at the 5% false detection rate threshold: adipocyte fatty acid binding protein (A-FABP) (regression coefficient 0.118, 95% CI (0.064, 0.173) *p* = 0.000024) and adrenomedullin (ADM) (regression coefficient 0.119, 95% CI (0.07, 0.168), *p* = 0.000002) The associations between all 88 proteins and leptin are shown in Supplementary Table 1.

In the replication cohort PIVUS, only A-FABP (*p* = 0.0003) but not ADM was associated with leptin (Table 2). The association between A-FABP and leptin were essentially similar after additional adjustment for other cardiovascular risk factors in both cohorts (Table 2) or when adding data on waist circumference or sagittal abdominal diameter to the multivariable models (data not shown). In exploratory analyses in PIVUS, the association between A-FABP and leptin was similar also in individuals without diabetes (n = 876, regression coefficient per SD increase 0.26, 95% CI 0.22–0.31, *p* < 0.001). Multiplicative interaction analyses in both CARDIPP and PIVUS suggest a stronger association between A-FABP and leptin in men as compared to women (CARDIPP *p* for interaction = 0.025 and PIVUS 0.026, Supplementary Table 2).

**Table 2:** Associations between leptin and proteins of interest in the discovery and replication cohorts CARDIPP and PIVUS: multivariate linear regression.

Variable	Beta 1	CI 1	P-value^1^	Beta 2	CI 2	P-value^2^
**CARDIPP**						
AMD	0.12	(0.07, 0.17)	0.000002	0.12	(0.08, 0.17)	0.0000006
A-FABP	0.12	(0.06, 0.17)	0.00002	0.11	(0.06, 0.16)	0.00009
**PIVUS**						
AMD	0.39	(− 0.06, 0.14)	0.47	0.04	(− 0.06, 0.15)	0.43
A-FABP	0.25	(0.12, 0.39)	0.0003	0.28	(0.14, 0.42)	0.0001

^1^Primary discovery analysis, adjusted for age, gender and BMI.

^2^Secondary analysis, adjusted also for systolic blood pressure, eGFR, LDL-, and HDL-cholesterol.

### Longitudinal analyses

During follow-up (mean 7.8 years), a total of 68 participants had an incident MACE event (incidence rate 1.4 per 100 person years follow-up). Multivariate Cox regression performed in the CARDIPP cohort showed that higher serum leptin levels were associated with the risk of MACE in a model adjusted for age, sex, BMI, systolic blood pressure, eGFR, LDL and HDL cholesterol [hazard ratio (HR) for each standard deviation increase in leptin level, 1.71, 95% CI 1.15–2.53, *p* = 0.007]. When adding A-FABP to the model, the association between leptin and MACE were attenuated slightly albeit still significant (HR 1.60, 95% CI 1.08–2.38, *p* = 0.02).

## Discussion

Using a discovery replication strategy and large-scale proteomics data, we identified consistent positive associations between leptin and A-FABP in two independent cohorts of patients with type 2 diabetes, an association which appeared stronger in men than in women. This association was similar after additional multivariable adjustment for cardiovascular risk factors. In longitudinal analyses, the association between leptin and MACE was slightly attenuated after adding A-FABP to the multivariate model which may indicate that A-FABP mediate some, but not all, of the link between leptin and CVD.

This is, to our knowledge, the first study to report associations between circulating levels of leptin and a multiplex proteomics assay. We are aware of only a few previous studies that have reported the association between circulating leptin and A-FABP. In one study, Reinehr et al. studied obese children before and after weight loss, showing a significant correlation between A-FABP and leptin and percentage of bodyfat, but not with other markers of the metabolic syndrome^21^. Another study in patients with lipodystrophy reported that A-FABP serum concentration correlated with gender and serum leptin as well as BMI^22^. Moreover, increased levels of both A-FABP and leptin has been shown in gestational diabetes mellitus, suggesting that A-FABP might be a contributor to the increased metabolic and cardiovascular risk of the disease^23,24^.

### Potential mechanisms

Adipose tissue has been studied extensively regarding its role in metabolic regulation through lipid signaling^25^. A-FABP, also known as adipocyte protein 2, aP2 or FABP4, is a fatty acid binding protein which can be found in white adipocytes and macrophages. In some studies, A-FABP has been identified as a circulating biomarker for metabolic syndrome, diabetes mellitus type 2, and cardiovascular events^26,27^. A-FABP is thought to be a central mediator of obesity-related CVD, and the production of A-FABP in adipocytes is thought to lead to insulin resistance and the expression of proinflammatory genes^28^. Tuncman et al. provided genetic support for the involvement of A-FABP in atherosclerosis in humans, where a reduction in A-FABP activity generated a metabolically favorable phenotype^29^ while Furuhashi et al. showed that inhibiting A-FABP is effective against severe atherosclerosis and type 2 diabetes in mice^30^. Human studies showed an association between A-FABP and coronary heart disease^31^ and has also been shown to be associated with peripheral arterial disease^32^. According to one study, A-FABP reduces the expression of leptin in mice adipocytes^33^, while another study in leptin deficient mice showed an impaired gene expression for lipid utilization for, amongst other proteins, A-FABP^34^. One study showed that A-FABP has a greater impact on atherosclerosis in women than in men, possibly related to the higher fat percentage in women^35^.

High serum leptin levels have been observed in patients with CHD^36^. There is a strong correlation between both circulating and adipose tissue levels of leptin and serum c-reactive protein (CRP) in obese women^37^. There is a known difference between leptin levels in men and women, where women have been shown to have about two times higher serum leptin levels than men at each level of BMI, a difference shown in both obese and non-obese^38,39^. Unfortunately, it is not possible to shed light on the underlying mechanisms for the seemingly stronger association between leptin and A-FABP in men in the present study. In clinical studies, correlations between leptin levels and established vascular risk factors, markers of impaired fibrinolysis, vascular dysfunction and inflammation have been shown^40^. A-FABP and leptin both act as interfaces between metabolic and inflammatory pathways, both expressed by adipocytes as well as being inflammatory mediators, where leptin can promote monocyte-macrophage-activation and A-FABP is produced by macrophages. Circulating levels of classical monocytes have been shown to be independently associated with cardiovascular events in both a cohort consisting of nearly a thousand coronary patients^41^, and in a randomly selected cohort of 700 subjects^42^. Monocytes in the plaque can give rise to foam cells, and those may in turn play an important role in plaque instability^43^.

Our study suggests that a small portion of the association between leptin and cardiovascular disease could be mediated via A-FABP, especially in men. However, our study cannot clarify whether leptin affects A-FABP or the other way around, and we can also not exclude the possibility that A-FABP is simply a confounder and not at all casually involved in the development of CVD. Leptin and A-FABP are both connected to macrophages in different stages, possibly connecting white adipose tissue with the inflammatory response.

### Clinical implications

Diabetes and CVD pose major disease burdens on the world’s population and understanding the mechanisms driving these diseases are of utmost importance. Proteomics analyses could be an important part in finding new associations between proteins and new drug targets. Whether A-FABP could be used as a target for intervention to influence leptin levels cannot be established in the present study.

### Strengths and limitations

Strengths of our study include the discovery/replication design, the longitudinal data and outcomes, and the consistent findings between different cohorts despite the small size of the cohorts. Strengths also include the use of registry data with high quality for mortality and morbidity. Limitations include the moderate sample size with limited power to detect weak associations, particularly in the replication cohort. The Olink assay does not give absolute concentrations of the proteins which may limit the generalizability and applicability of our results to other cohorts. Generalizability is limited to middle-aged to elderly Caucasian adults (55–70 years of age) with type 2 diabetes. BMI is not an ideal measurement of body fat, but associations were similar when adding waist circumference of sagittal abdominal diameter to the model.

## Conclusions

Our proteomics-based analysis identified a consistent association between leptin and A-FABP. Additional studies are warranted to validate our findings and define clinical utility, in the hopes of improving strategies for CVD prevention.

## Supplementary information

Supplementary tables.

## Data Availability

The datasets used and/or analysed during the current study are available from the corresponding author on reasonable request.

## Abbreviations

ADM: Adrenomedullin
A-FABP: Adipocyte fatty acid binding protein
BMI: Body mass index
CARDIPP: Cardiovascular risk factors in patients with diabetes—a prospective study in primary care
CHD: Coronary heart disease
CRP: C-reactive protein
CVD: Cardiovascular disease
HDL: High density lipoprotein
HR: Hazard ratio
LDL: Low density lipoprotein
LOD: Limit of detection
MACE: Major adverse cardiac events
PCR: Polymerase chain reaction
PIVUS: Prospective Investigation of the Vasculature in Uppsala Seniors
RIA: Radioimmunoassay
